# XRCC1 Deficiency Sensitizes Human Lung Epithelial Cells to Genotoxicity by Crocidolite Asbestos and Libby Amphibole

**DOI:** 10.1289/ehp.1002312

**Published:** 2010-08-12

**Authors:** Jodie R. Pietruska, Tatiana Johnston, Anatoly Zhitkovich, Agnes B. Kane

**Affiliations:** Department of Pathology and Laboratory Medicine, Brown University, Providence, Rhode Island, USA

**Keywords:** asbestos, crocidolite, DNA breaks, DNA repair, Libby amphibole, micronuclei, nuclear buds, XRCC1

## Abstract

Background: Asbestos induces DNA and chromosomal damage, but the DNA repair pathways protecting human cells against its genotoxicity are largely unknown. Polymorphisms in *XRCC1* have been associated with altered susceptibility to asbestos-related diseases. However, it is unclear whether oxidative DNA damage repaired by XRCC1 contributes to asbestos-induced chromosomal damage.

Objectives: We sought to examine the importance of XRCC1 in protection against genotoxic effects of crocidolite and Libby amphibole asbestos.

Methods: We developed a genetic model of XRCC1 deficiency in human lung epithelial H460 cells and evaluated genotoxic responses to carcinogenic fibers (crocidolite asbestos, Libby amphibole) and nongenotoxic materials (wollastonite, titanium dioxide).

Results: XRCC1 knockdown sensitized cells to the clastogenic and cytotoxic effects of oxidants [hydrogen peroxide (H_2_O_2_), bleomycin] but not to the nonoxidant paclitaxel. XRCC1 knockdown strongly enhanced genotoxicity of amphibole fibers as evidenced by elevated formation of clastogenic micronuclei. Crocidolite induced primarily clastogenic micronuclei, whereas Libby amphibole induced both clastogenic and aneugenic micronuclei. Crocidolite and bleomycin were potent inducers of nuclear buds, which were enhanced by XRCC1 deficiency. Libby amphibole and H_2_O_2_ did not induce nuclear buds, irrespective of XRCC1 status. Crocidolite and Libby amphibole similarly activated the p53 pathway.

Conclusions: Oxidative DNA damage repaired by XRCC1 (oxidized bases, single-strand breaks) is a major cause of chromosomal breaks induced by crocidolite and Libby amphibole. Nuclear buds are a novel biomarker of genetic damage induced by exposure to crocidolite asbestos, which we suggest are associated with clustered DNA damage. These results provide mechanistic evidence for the epidemiological association between *XRCC1* polymorphisms and susceptibility to asbestos-related disease.

## Editor's Summary

Pathways involved in the repair of asbestos-induced DNA damage are largely unknown, but *XRCC1* polymorphisms that may influence the efficacy of DNA repair have been associated with susceptibility to asbestos-related diseases. Pietruska et al. (p. 1707) examined the role of XRCC1 in genotoxic effects of crocidolite and Libby amphibole asbestos in normal and XRCC1-deficient human lung epithelial H460 cells. The authors report that oxidative DNA damage, including oxidized bases and single-strand breaks, was increased following asbestos exposure in XRCC1-deficient cells compared with control cells, and was a major cause of chromosomal breaks induced by crocidolite and Libby amphibole. Nuclear buds, a possible marker of clustered DNA damage, were induced by crocidolite asbestos but not Libby amphibole. The authors conclude that their findings provide mechanistic support for epidemiologic evidence linking *XRCC1* polymorphisms and susceptibility to asbestos-related disease.

Occupational and environmental exposure to asbestos is strongly associated with the development of asbestosis, lung cancer, and malignant mesothelioma. Asbestos acts in part as a genotoxic carcinogen, inducing mutations and gross chromosomal damage (Dopp et al. 1995; Shukla et al. 2003). Asbestos induces the formation of reactive oxygen species (ROS), reactive nitrogen species (Shukla et al. 2003), DNA strand breaks (Jaurand 1997), and oxidized bases, including the premutagenic lesion 8-hydroxy-2´-deoxyguanosine (8-oxodG) (Unfried et al. 2002; Xu et al. 1999). The genotoxic effects of asbestos fibers are largely attributed to the participation of surface redox-active iron in Fenton reactions that generate ROS (Upadhyay and Kamp 2003). The presence of surface iron correlates with induction of DNA strand breaks in acellular systems, and asbestos-induced DNA damage is prevented by iron chelators and antioxidants in cultured cells (Upadhyay and Kamp 2003).

Although the DNA repair pathways activated by asbestos are largely unknown, the spectrum of DNA damage induced by asbestos suggests a role for single-strand breaks (SSBs) in asbestos-induced genotoxicity. *XRCC1* (X-ray repair cross-complementing protein 1) is essential for successful SSB repair (Caldecott 2003). SSB resolution is critical for viability, because *XRCC1*-knockout mice die *in utero* due to accumulation of endogenous DNA damage (Tebbs et al. 1999). XRCC1 has no known enzymatic activity but is thought to function as a molecular scaffold to recruit and stabilize DNA repair proteins at the sites of SSBs. XRCC1 also plays a role in removal of oxidized DNA bases, including 8-oxodG, via base excision repair (BER) (Tudek 2007).

*XRCC1* mutations have not been identified in human tumors, but *XRCC1* single-nucleotide polymorphisms (SNPs) may influence cancer susceptibility by altering the efficacy of DNA repair. Polymorphic *XRCC1* variants may also alter susceptibility to asbestos-induced diseases, because substitution of glutamine for arginine at codon 399 (Arg399Gln) has been associated with elevated DNA damage in peripheral blood lymphocytes of asbestos-exposed workers (Zhao et al. 2006) and elevated risk of asbestosis, lung cancer, and mesothelioma (Dianzani et al. 2006; Neri et al. 2008). Although the mechanistic basis for these associations is unknown, individuals carrying polymorphic *XRCC1* variants may exhibit reduced DNA repair activity and thus may be predisposed to the accumulation of DNA damage.

To date, no genetic model has been developed to address the role of oxidative damage in asbestos-induced genotoxicity or the role of XRCC1 in protecting human cells from asbestos-induced damage. It is plausible that XRCC1 is involved in repair of asbestos- induced DNA damage because asbestos induces multiple DNA lesions that are repaired by XRCC1-dependent pathways. Additionally, two types of chromosomal damage induced by asbestos—micronuclei and sister chromatid exchanges—are specifically elevated by XRCC1 deficiency in Chinese hamster ovary (CHO) cells (Qu et al. 2005; Thompson et al. 1982). Last, asbestos increases activity of key BER enzymes *in vitro*, including poly(ADP-ribose) polymerase (PARP) and AP endonuclease 1 (Fung et al. 1998; Kamp et al. 2001).

In order to test whether XRCC1 protects lung epithelial cells from asbestos-induced DNA damage, we developed a genetic model of XRCC1 deficiency in human H460 cells. We evaluated the effect of XRCC1 knockdown on genotoxicity using noncarcinogenic fibers (wollastonite) and particles [titanium dioxide (TiO_2_)] and two types of amphibole asbestos, International Union Against Cancer (UICC) crocidolite and Libby amphibole. Libby amphibole is a mixture of noncommercial amphiboles obtained from the vermiculite mine in Libby, Montana. Occupational and environmental exposure to Libby amphibole is associated with development of asbestos-related diseases among workers and residents of Libby (Amandus and Wheeler 1987; McDonald et al. 2004; Sullivan 2007). Unlike crocidolite, data are limited regarding the genotoxic potential of Libby amphibole (Blake et al. 2007).

## Materials and Methods

*Cell culture and treatments.* We obtained H460 human lung epithelial cells from the American Type Culture Collection (ATCC, Manassas, VA) and cultured them in RPMI 1640/10% fetal bovine serum/1% penicillin/streptomycin in a humidified atmosphere (6% CO_2_/94% air). NYAD 1250 wollastonite was a generous gift from NYCO Minerals, Inc. (Willsboro, NY). We obtained TiO_2_ (anatase, 325-mesh) from Sigma-Aldrich (St. Louis, MO), and crocidolite asbestos fibers from stocks originally prepared and characterized by the UICC (Timbrell 1970) were purchased from Duke Scientific (Palo Alto, CA). The U.S. Geological Survey provided the Libby amphibole (“Libby 6-mix”). The size distribution of fiber samples was determined by transmission electron microscopy as described by Moalli et al. (1987) [see Supplemental Material, Table 1 (doi:10.1289/ehp.1002312)]. We baked TiO_2_, wollastonite, and both amphibole fibers at 250°C overnight to inactivate endotoxin contaminants (if present), and we sterilized them by autoclaving in phosphate-buffered saline (PBS). Before use, we dispersed materials for 1 hr (wollastonite, TiO_2_, crocidolite) or 2 hr (Libby amphibole) by sonication.

*Toluidine blue staining of fiber internalization.* We plated H460 cells at 12,500 cells/cm^2^ in 100-mm dishes and treated them with 5 µg/cm^2^ fibers or TiO_2_ particles in serum-free medium for 6 hr. Cells were fixed in 2% glutaraldehyde/0.1 M sodium cacodylate buffer and stored in cacodylate buffer/8% sucrose at 4°C. Samples were subsequently treated with 1% osmium tetroxide and dehydrated through a series of graded ethanols. Samples were infiltrated overnight with 100% ethanol and Spurr embedding medium (1:1; Electron Microscopy Sciences, Hatfield, PA), infiltrated with fresh Spurr resin, embedded in molds, and polymerized at 60°C. Blocks were sectioned at 0.5 µm on a Reichert ultramicrotome (Leica, Richmond, IL), and sections were stained with toluidine blue.

*Short-hairpin RNA knockdown.* We generated knockdown populations of H460 cells as described elsewhere (Reynolds et al. 2004). The oligonucleotides containing targeting sequences (shown in uppercase) for firefly luciferase [short-hairpin luciferase (shLuc) controls] and human *XRCC1* (shXRCC1 knockdown) were as follows: 5´-gatccccGCGACCAACGCCTTGATTGttcaagagaCAATCAAGGCGTTGGTCGCtttttggaa-3´  for luciferase; and 5´-gatccccAGGGGAAGAGGAAGTTGGATttcaagagaATCCAACTTCCTCTTCCCTttttta-3´ for *XRCC1*. Knockdown cells were maintained in 1.5 µg/mL  puromycin and used for experiments within 7 days of infection.

*PCR/RFLP analysis for XRCC1 SNPs.* We isolated genomic DNA from H460 cells using the DNeasy kit (Qiagen, Valencia, CA) according to the manufacturer’s instructions. Polymorphisms in codons 194, 280, and 399 were assessed using polymerase chain reaction (PCR)/restriction fragment length polymorphism (RFLP) analysis with the following primers: 194 forward, 5´-ggttccgtgtgaaggaggagga-3´; 194 reverse, 5´-cgagtctaggtctcaaccctactcact-3´; 280 forward, 5-ggggttgacccccagtggtgctaa-3´; 280 reverse, 5´-ggctccgaccacctgtgttctc-3´; 399 forward, 5´-ttgtgctttctctgtgtcca-3´; 399 reverse, 5´-tcctccagccttttctgata-3´.

Each 50-µL PCR reaction contained 625 ng genomic DNA, 200 nM dNTP, 2 mM MgSO_4_, 1 U High-Fidelity Platinum Taq polymerase (Invitrogen, Carlsbad, CA) and 0.2 µM each primer in 1× PCR buffer. The PCR conditions were as follows: initial denaturation (95°C, 30 sec), 30 cycles of denaturation (95°C, 30 sec), annealing [60°C, 30 sec (codons 194 and 280) or 58°C, 30 sec (codon 399)], elongation (72°C, 90 sec), and a 10-min elongation at 72°C. PCR products were digested with *Pvu*II (codon 194), *Rsa*I (codon 280), or *Hpa*II (codon 399) and resolved on a 4% agarose gel stained with ethidium bromide.

*Cell proliferation.* To establish a range of sublethal doses for the fibers and TiO_2_ particles, we plated shLuc and shXRCC1 H460 cells at 12,500 cells/cm^2^ into 96-well plates, treated them with 0–20 µg/cm^2^ fibers in serum-free medium for 24 hr, and restored the serum concentration to 10% for the remainder of the experiment (24–96 hr total). We assessed cell proliferation using the CyQuant Cell Proliferation Assay kit (Invitrogen) according to the manufacturer’s instructions for DNA content analysis. A standard curve based on H460 cell number was used to demonstrate the linear relationship between cell number and fluorescence (*R*^2^ = 0.993; data not shown). Proliferation was expressed as percentage of cell growth after treatment relative to cell growth in untreated shLuc or shXRCC1 H460 cells at the beginning of the experiment.

*Clonogenic survival.* To functionally validate XRCC1 knockdown, we challenged H460 cells with hydrogen peroxide (H_2_O_2_) and bleomycin (known oxidants) and with paclitaxel (a nonoxidant). shXRCC1 and shLuc H460 cells were plated at 500 cells/60-mm dish and treated with 0–60 µM H_2_O_2_ or 0–10 µg/mL bleomycin in serum-free medium for 1 and 3 hr, respectively, or with 0–5 nM paclitaxel in normal growth medium for 24 hr. Cells were rinsed with serum-free medium and cultured in normal growth medium for 7 days prior to staining with Giemsa. We scored colonies in four replicate dishes, and scores are expressed as the percentage of survival relative to a mock-treated control.

*XRCC1 immunofluorescence.* We plated uninfected H460 cells and untreated shLuc and shXRCC1 H460 cells onto 18-mm glass coverslips at 12,500 cells/cm^2^; cells were fixed in 2% paraformaldehyde/PBS, permeabilized in 1% Triton X-100/PBS, and blocked in 5% normal goat serum/0.1% bovine serum albumin/0.1% Tween-20/PBS. Coverslips were incubated with XRCC1 antibody (Abcam, Cambridge, MA) and AlexaFluor 488 goat anti-mouse IgG (Invitrogen), mounted in Vectashield/DAPI (Vector Labs, Burlingame, CA), and visualized using a Nikon fluorescence microscope. Images were obtained using Spot software (version 3.5.8; SPOT Imaging Solutions, Sterling Heights, MI).

*Micronucleus formation/CREST (immunofluorescent antikinetochore) staining.* We plated shLuc and shXRCC1 H460 cells onto 18-mm glass coverslips at 12,500 cells/cm^2^ and treated the cells with H_2_O_2_, bleomycin, or paclitaxel (as described under clonogenic survival) or with 5 µg/cm^2^ fibers or TiO_2_ particles (as described for cell proliferation assays). After treatment, the cells were fixed in 2% paraformaldehyde/PBS, permeabilized in 1% Triton X-100/PBS, and blocked with 5% normal goat serum/PBS. Coverslips were incubated with anti-centromere antibody [CREST serum; University of California (UC)–Davis/National Institute of Neurological Disorders and Stroke/National Institute of Mental Health NeuroMab Facility, UC Davis, Davis, CA] and AlexaFluor 488 anti-human IgG (Invitrogen) and mounted in Vectashield/DAPI. We scored 1,000 interphase nuclei per coverslip in triplicate for micronuclei or nuclear buds. Micronuclei were scored as centromere negative (clastogenic) or centromere positive (aneugenic) based on the presence of CREST staining, which recognizes centromere proteins within the kinetochore of each chromosome, within each micronucleus. We performed the micronucleus/CREST assay in the absence of a cytokinesis block because recent work has shown equivalent micronucleus formation in the presence and absence of cytochalasin B (Lorge et al. 2006); cytochalasin B inhibits internalization of asbestos fibers as well as their genotoxic and cytotoxic effects (Liu et al. 2000).

*Western blot analysis.* Cells were plated at 12,500 cells/cm^2^ in 100-mm dishes, treated as indicated, and lysed as described elsewhere (Pietruska and Kane 2007). We immunoblotted with antibodies against XRCC1 (Abcam), phosphorylated p53 (Ser15; Cell Signaling Technology, Danvers, MA), p53 (DO-1; Santa Cruz Biotechnology, Santa Cruz, CA), p21 (SX118; BD Pharmingen, San Diego, CA), or β-actin (Sigma-Aldrich). Blots were visualized using Amersham enhanced chemiluminescence (GE Healthcare, Piscataway, NJ).

*Statistical analysis.* We performed one-way analysis of variance (ANOVA) and the Holm-Sidak method for pairwise comparison using SigmaStat software (SPSS Inc., Chicago, IL).

## Results

**Figure 1:**
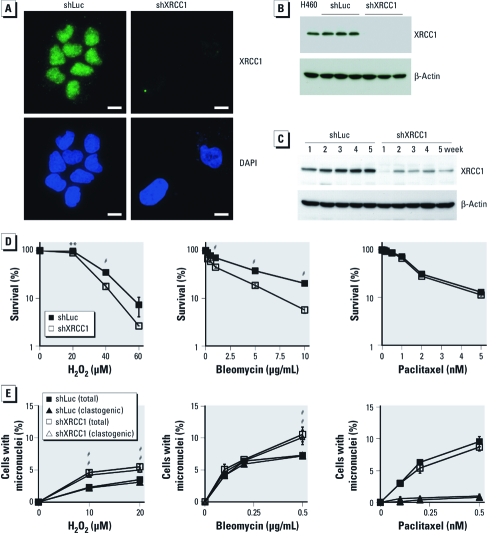
Internalization of mineral particles and fibers in H460 cells. (*A*) Particle and fiber internalization occurred within 6 hr. Arrowheads indicate intracellular wollastonite, TiO_2_, crocidolite, and Libby amphibole; bars = 20 µm. (*B*) PCR/RFLP analysis of *XRCC1* codons 194, 280, and 399. H460 cells are wild-type (WT) for *XRCC1* at codon 399, at codon 194 (indicated by the absence of a *Pvu*II restriction site), and at codon 399 (indicated by the presence of a *Hpa*II restriction site). (*C*) Predicted restriction fragment sizes for WT and polymorphic sequences. H460 cells are homozygous for Arg280His, indicated by the presence of 201 and 660 bp restriction fragments.

*Lung epithelial cells internalize particles and mineral fibers.* We used the H460 human lung epithelial cell line to investigate the effects of XRCC1 deficiency in a cell type relevant to asbestos carcinogenesis. H460 cells have an intact DNA damage response (Zhang et al. 2006) and accurately recapitulate the responses of primary human cells to genotoxic stress (Reynolds et al. 2009; Reynolds and Zhitkovich 2007). Because previous studies indicated that the oxidative, clastogenic, and cytotoxic effects of asbestos fibers require internalization (Liu et al. 2000), we verified that H460 cells internalized particles and fibers. Fiber internalization occurred within 6 hr of exposure, and nontoxic fibers and particles (wollastonite, TiO_2_) and carcinogenic fibers (crocidolite, Libby amphibole) all appeared to localize within intracellular vacuoles (Figure 1A, arrowheads).

**Figure 2:**
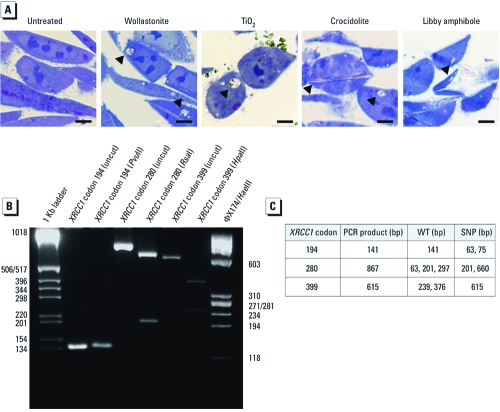
XRCC1 deficiency sensitizes H460 cells to oxidative damage. (*A*) XRCC1 immunofluorescence in shLuc and shXRCC1 cells. XRCC1 (green) localizes to the nucleus of shLuc cells (used as a proxy for uninfected H460 cells), and shXRCC1 cells show uniform loss of XRCC1 expression (as shown by the lack of staining in DAPI-stained nuclei); bars = 50 µm. (*B*) Western blot analysis of XRCC1 expression before and after infection with shRNA targeting firefly luciferase (shLuc) or human XRCC1 (shXRCC1). Three independent infections are shown. (*C*) Western blot analysis of XRCC1 expression 0–5 weeks after infection; XRCC1 expression was partially restored within 2 weeks of infection. β-Actin was used as a loading control for Western blots. (*D*) XRCC1 deficiency reduces clonogenic survival of H460 cells exposed to H_2_O_2_ or bleomycin but not paclitaxel. Data shown are mean ± SE of four replicates. (*E*) XRCC1 deficiency enhances micronucleus formation 48 hr after exposure to H_2_O_2_ or bleomycin but not paclitaxel. Data shown are mean ± SE of three replicates, for which the percentage of spontaneous micronuclei has been subtracted. **p < 0.01, and ^#^*p* < 0.001 compared with shLuc by one-way ANOVA.

*Evaluation of common SNPs of* XRCC1. Numerous *XRCC1* SNPs have been identified, and polymorphisms affecting binding of XRCC1 to DNA repair proteins may result in suboptimal repair. The three most extensively studied coding *XRCC1* SNPs are Arg194Trp, Arg280His, and Arg399Gln. In particular, Arg399Gln is associated with elevated risk of lung cancer and mesothelioma in asbestos- exposed workers (Dianzani et al. 2006; Neri et al. 2008). Codon 399 is located in the BRCT I domain of XRCC1 required for PARP binding and SSB repair. Arg399Gln is predicted to generate conformational changes within this domain (Monaco et al. 2007), which may impair the ability of XRCC1 to interact with and stabilize PARP at SSBs, thereby reducing repair efficiency. Using PCR/RFLP analysis, we found that H460 cells do not contain the Arg194Trp polymorphism, indicated by the lack of a *Pvu*II restriction site at codon 194 (Figure 1B,C). H460 cells also do not contain the Arg399Gln polymorphism because enzymatic digest with *Hpa*II generates restriction fragments consistent with the wild-type sequence at codon 399. H460 cells do contain the Arg280His polymorphism, as enzymatic digest with *Rsa*I generates fragments consistent with the polymorphic sequence (Figure 1B,C). The presence of this polymorphism, located within the XRCC1 nuclear localization signal, does not appear to affect XRCC1 nuclear localization because XRCC1 is correctly localized to the nucleus (Figure 2A).

*XRCC1 deficiency sensitizes to oxidant-induced damage and toxicity.* Short-hairpin RNA (shRNA) targeting XRCC1 in H460 cells (shXRCC1) induced efficient knockdown of XRCC1 relative to uninfected cells or control shLuc cells (Figure 2B). Compared with control, XRCC1-knockdown cells exhibited uniform XRCC1 loss (Figure 2A), although XRCC1 was partially reexpressed within 2 weeks postinfection (Figure 2C), despite being cultured in the presence of the selecting antibiotic puromycin. Based on this finding and on previous studies showing reversal of the phenotypic effects of XRCC1 deletion by low-level XRCC1 expression (Caldecott and Thompson 1994; Tebbs et al. 2003), we began all experiments within 1 week of infection.

To functionally validate XRCC1 knockdown, we evaluated clonogenic survival of shLuc and shXRCC1 cells exposed to subcytoxic doses of two oxidants, H_2_O_2_ and bleomycin. Compared with controls, XRCC1 knockdown decreased clonogenic survival after exposure to H_2_O_2_ or bleomycin (Figure 2D). In contrast, XRCC1 deficiency had no effect on clonogenic survival in cells exposed to paclitaxel, a microtubule depolymerization inhibitor whose genotoxicity and cytotoxicity are oxidant independent.

To test whether XRCC1 knockdown sensitized H460 cells to chromosomal damage, we used CREST immunostaining, which recognizes centromere proteins within the kinetochore of each chromosome. The micronucleus/ CREST assay allows determination of the total micronucleus frequency, the frequency of centromere-negative (clastogenic) micronuclei arising from DNA breaks, and the frequency of centromere-positive (aneugenic) micronuclei arising from chromosome loss during mitosis. Relative to shLuc controls, XRCC1 knockdown increased the frequency of spontaneous micronuclei from 1.4% to 3.8% (*p* < 0.001) and spontaneous clastogenic micronuclei from 1.2% to 3.4% [*p* < 0.001; see Supplemental Material, Figure 1A (doi:10.1289/ehp.1002312)], which is consistent with a previous report of increased spontaneous micronuclei formation in *Xrcc1*-null cells (Qu et al. 2005). Treatment of both control (shLuc) and XRCC1-deficient (shXRCC1) cells with H_2_O_2_ induced a dose-dependent increase in micronuclei, nearly all of which were clastogenic (Figure 2E). However, the percentage of cells with H_2_O_2_-induced micronuclei, both total and clastogenic, was increased in XRCC1 knockdown cells compared with controls. Likewise, bleomycin induced a dose- dependent increase in micronucleus formation in control and XRCC1 knockdown cells, producing almost exclusively clastogenic micronuclei (Figure 2E), with higher percentages of cells with micronuclei (total and clastogenic) in shXRCC1 cells at bleomycin doses between 0.2 µg/mL and 0.5 µg/mL. However, XRCC1 deficiency did not enhance formation of paclitaxel-induced micronuclei, most of which were centromere positive (aneugenic) (Figure 2E). Taken together, these results confirm that XRCC1 deficiency specifically sensitizes H460 cells to oxidant-induced damage.

**Figure 3:**
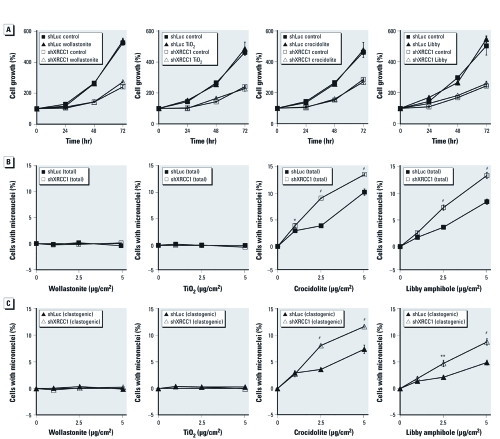
XRCC1 deficiency enhances chromosomal damage induced by crocidolite asbestos and Libby amphibole. (*A*) Exposure to fibers or particles does not inhibit growth of H460 cells expressing shLuc or shXRCC1 (XRCC1-knockdown cells). Data shown are mean ± SE of four replicates. (*B* and *C*) XRCC1 deficiency increases the percentage of cells with total micronuclei (*B*) and clastogenic micronuclei (*C*) after exposure to carcinogenic fibers, shown by micronucleus formation and CREST immunostaining 48 hr (shLuc) or 72 hr (shXRCC1) after exposure to 5 µg/cm^2^ fibers, at which time cells have completed one cell division. Data shown are mean ± SE of three replicates, in which the percentage of spontaneous micronuclei has been subtracted. *p < 0.05, **p < 0.01, and ^#^*p* < 0.001, compared with shLuc by one-way ANOVA.

*XRCC1 deficiency enhances genotoxicity induced by asbestos fibers.* Because XRCC1 deficiency sensitized to clastogenicity induced by known oxidants (H_2_O_2_ and bleomycin), we next asked whether it would enhance clastogenic damage induced by asbestos fibers. To establish a subcytotoxic dose range of fibers, we evaluated cell proliferation during a 72-hr exposure to wollastonite, TiO_2_, crocidolite, or Libby amphibole. We chose a dose of 5 µg/cm^2^ because micronucleus induction in parental H460 cells plateaued at doses of crocidolite or Libby amphibole > 5 µg/cm^2^  [see Supplemental Material, Figure 1B (doi:10.1289/ehp.1002312)] and also because of activation of the p53 pathway after exposure to amphibole fibers at 5 µg/cm^2^  (see Supplemental Material, Figure 2). We observed a pronounced reduction in cell proliferation in XRCC1-knockdown versus control cells (Figure 3A), consistent with a report of slower growth rates in *Xrcc1*-null mouse embryos (Tebbs et al. 1999). However, proliferation was not affected by exposure to nontoxic fibers, TiO_2_ particles, or asbestos fibers in control or XRCC1-knockdown cells (Figure 3A).

Because micronucleus formation requires passage through mitosis, we scored micronuclei in control and XRCC1-knockdown cells after 48-hr and 72-hr exposures, respectively, at which times both cell lines had completed one population doubling. The percentage of H460 cells with fiber-induced micronuclei was similar at both time points [see Supplemental Material, Figure 1B (doi:10.1289/ehp.1002312)], indicating that we were not underestimating differences in micronuclei by normalizing to one population doubling. As expected, wollastonite and TiO_2_ did not induce micronuclei in control or XRCC1-knockdown cells (Figure 3B). In contrast, crocidolite asbestos and Libby amphibole induced dose-dependent increases in micronucleus formation in both control and XRCC1-knockdown cells. However, the proportion of cells with crocidolite- induced micronuclei (total and clastogenic; Figure 3B,C) and the proportion of crocidolite- induced micronuclei that were clastogenic (mean ± SE, 86.0 ± 1.2% vs. 71.6 ± 3.4% at 5 µg/cm^2^; *p* < 0.05) were increased in XRCC1-deficient cells compared with control cells. Induction of total and clastogenic micronuclei by Libby amphibole also was enhanced in XRCC1-deficient cells compared with controls (Figure 3B,C), along with the proportion of clastogenic micronuclei (mean ± SE, 65.1 ± 2.2% vs. 57.2 ± 2.2% at 5 µg/cm^2^; *p* < 0.05). However, the proportion of clastogenic micronuclei induced by Libby amphibole was lower than that observed after exposure to crocidolite (*p* < 0.05 for shLuc and shXRCC1 cells). Taken together, these results indicate that XRCC1 deficiency sensitizes cells to the genotoxic and clastogenic effects of crocidolite and Libby amphibole.

Although XRCC1 knockdown did not inhibit proliferation of H460 cells at a fiber dose of 5 µg/cm^2^, it was possible that higher doses would inhibit growth. To investigate this possibility, we exposed control and XRCC1-knockdown cells to 5–20 µg/cm^2^ fibers for 72 hr. Although crocidolite asbestos and, to a lesser extent, Libby amphibole decreased cell proliferation in a dose-dependent manner, this was not enhanced by XRCC1 deficiency [see Supplemental Material, Figure 3A (doi:10.1289/ehp.1002312)]. Extending the duration of fiber exposure to 96 hr further increased the growth inhibition by crocidolite asbestos and Libby amphibole; however, this was not further enhanced by XRCC1 deficiency (see Supplemental Material, Figure 3B). These results indicate that, although XRCC1 sensitizes to genotoxicity induced by carcinogenic fibers, it does not potentiate their growth inhibitory effects.

**Figure 4:**
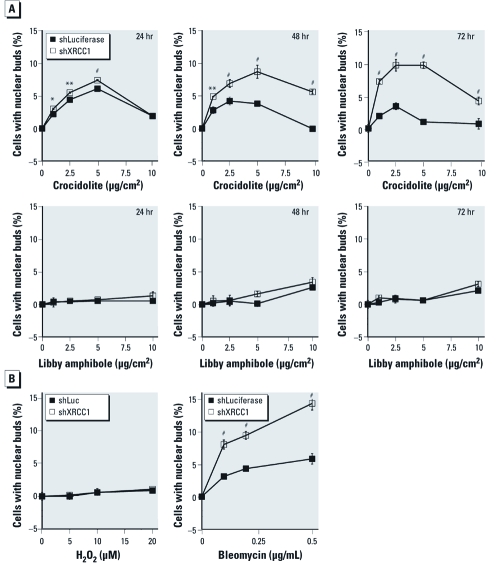
XRCC1 deficiency enhances formation of nuclear buds by bleomycin and crocidolite asbestos. (*A*) Time course of bud formation by carcinogenic fibers; shLuc cells and shXRCC1 cells were scored for nuclear buds after 24–72 hr exposures to crocidolite or Libby amphibole. (*B*) Induction of buds by bleomycin but not H_2_O_2_; shLuc cells and shXRCC1 cells were scored for nuclear buds 48 hr after exposure to H_2_O_2_ or bleomycin. Data shown are mean ± SE of three replicates after subtraction of spontaneous nuclear buds. *p < 0.05, **p < 0.01, and ^#^*p* < 0.001, compared with shLuc by one-way ANOVA.

*XRCC1 deficiency enhances formation of nuclear buds.* Although micronuclei are widely used indicators of genomic damage, nuclear buds are another, less commonly studied, marker of genotoxicity. In contrast to micronuclei, which are detached from the main nucleus, nuclear buds remain attached to the nucleus through a thin nucleoplasmic stalk (Fenech 2007) [see Supplemental Material, Figure 4A (doi:10.1289/ehp.1002312)]. XRCC1 deficiency increased the frequency of spontaneous nuclear buds from 4.9% to 9.1% (*p* < 0.05 compared with shLuc control cells); therefore, we expressed the data after background subtraction. Because it was possible that the kinetics of nuclear bud formation were different from that of micronuclei and potentially did not depend on cell division, we compared bud formation after 24, 48, and 72 hr of exposure to fibers at doses of 2.5, 5.0, and 10 µg/cm^2^ (Figure 4A). Wollastonite and TiO_2_ did not induce nuclear bud formation (see Supplemental Material, Figure 4B). Crocidolite asbestos induced bud formation that reached a maximum at 2.5–5 µg/cm^2^ (Figure 4A) and was enhanced in XRCC1-deficient cells compared with controls at all doses. However, Libby amphibole did not induce significant bud formation in control or XRCC1-deficient cells (Figure 4A). H_2_O_2_, a potent inducer of SSBs, also did not induce bud formation in control or XRCC1-deficient cells. In contrast, exposure to bleomycin, a radiomimetic chemotherapeutic agent that induces substantial double-strand breaks (DSBs) in addition to SSBs, caused a dose-dependent increase in nuclear buds in control cells that was significantly enhanced by XRCC1 knockdown (Figure 4B). Taken together, our results suggest that XRCC1 protects H460 cells from genotoxicity induced by crocidolite asbestos or Libby amphibole.

## Discussion

In this study we developed a genetic model of XRCC1 deficiency in human lung epithelial cells that is highly relevant to asbestos carcinogenesis and offers several advantages over other experimental models, including a low level of spontaneous micronuclei and sensitivity to genotoxicants at low doses (Reynolds et al. 2009; Reynolds and Zhitkovich 2007). Genetic models of DNA repair deficiency allow us to directly evaluate the contribution of particular DNA lesions to fiber-induced genotoxicity in the absence of downstream effects on multiple cellular stress response pathways, as can happen with antioxidant-based approaches.

*Impact of XRCC1 on asbestos genotoxicity.* Crocidolite and Libby amphibole asbestos induced dose-dependent increases in micronuclei in control cells, which were enhanced by XRCC1 deficiency. Interestingly, the relative proportions of clastogenic micronuclei varied considerably because crocidolite induced primarily clastogenic micronuclei, whereas Libby amphibole induced both aneugenic and clastogenic damage. We also observed nuclear buds in H460 cells treated with bleomycin or with crocidolite, but not with Libby amphibole or nongenotoxic fibers or particulates. Nuclear buds containing amplified DNA have been described in tumor cells (Shimizu et al. 1998) and in folate-deprived lymphocytes (Lindberg et al. 2007), but this is the first report of nuclear bud formation in response to asbestos fibers. The mechanism of nuclear bud formation is unclear, although nuclear buds formed during interphase may represent precursors to micronuclei (Serrano-Garcia and Montero-Montoya 2001); however, our observations that H_2_O_2_ and Libby amphibole induce micronuclei but not nuclear buds are inconsistent with this hypothesis. Nuclear buds may also form as part of a mechanism to remove excess nuclear DNA, or from broken anaphase bridges (Gisselsson et al. 2001).

Our findings in H_2_O_2_- and bleomycin-treated cells suggest that the formation of nuclear buds is associated with clustered DNA damage. Although H_2_O_2_ induces primarily SSBs that are randomly distributed within DNA, bleomycin produces SSBs, DSBs, and clustered DNA damage (Regulus et al. 2007). Compared with H_2_O_2_, which did not induce significant bud formation, bleomycin induced a dose-dependent increase in nuclear buds, which was enhanced by XRCC1 deficiency, suggesting that nuclear buds could arise because of clustered DNA damage. Crocidolite, like bleomycin, induced nuclear bud formation that was enhanced by XRCC1 deficiency, suggesting that crocidolite, but not Libby amphibole, has the potential to induce clustered DNA damage.

*Differential genotoxicity of crocidolite asbestos and Libby amphibole.* Although both crocidolite and Libby are amphiboles, their distinct physicochemical properties may influence the types of damage they produce. Clastogenic micronuclei arise from chromosome breaks, and the ability of asbestos to generate DNA breaks is strongly linked to the presence of fiber-associated iron and ROS generation (Shukla et al. 2003). Crocidolite asbestos contains up to 27% iron by weight (Timbrell 1970), whereas Libby amphibole contains approximately 5% iron by weight (Meeker et al. 2003). Therefore, the different proportions of clastogenic micronuclei generated by crocidolite asbestos and Libby amphibole may reflect their different iron contents.

In addition to differences in bulk iron content, crocidolite and Libby amphibole differ greatly in amounts of bioavailable redox-active iron, which is responsible for ROS generation (Hardy and Aust 1995). Using an assay to assess iron bioavailability, we observed that iron mobilization was 20 times higher per milligram of crocidolite versus Libby amphibole (Kulaots I, Vaslet C, Kane AB, unpublished data). Iron bioavailability and redox activity are also related to surface area, which is 9.1 m^2^/g for crocidolite compared with 5.3 m^2^/g for Libby amphibole. Finally, these amphiboles differ in homogeneity, geometry, and fiber size distribution. UICC crocidolite asbestos is a homogeneous sample ranging from 30 to 500 nm in diameter and up to 20 µm in length. In contrast, Libby amphibole is a mixture of six amphiboles, mostly winchite (84%), richterite (11%), and tremolite (6%), including prismatic crystals, acicular fragments, and bundles of fibrils approximately 100 nm to 1 µm in diameter with lengths up to 20 µm (Meeker et al. 2003). The heterogeneity of Libby amphibole, in addition to its lower bioavailable iron, may be associated with its lower clastogenicity compared with crocidolite in the present study. Although the mechanistic basis for these differences remains to be determined, these results are the first to indicate that Libby amphibole has genotoxic potential in a cell type relevant to malignant transformation and that, in this model system, Libby amphibole induces both clastogenic and aneugenic damage, whereas crocidolite induces primarily clastogenic damage.

In contrast to genotoxicity, XRCC1 deficiency did not affect growth inhibition by crocidolite or Libby amphibole. H460 cells are not resistant to asbestos-induced growth inhibition because higher doses and longer  exposures reduced cell proliferation [see Supplemental Material, Figure 2 (doi:10.1289/ehp.1002312)]. XRCC1 appeared to protect cells from asbestos-induced genotoxicity in this model, but the pathways responsible for the growth-inhibitory effects of fibers are XRCC1 independent and may involve multiple forms of damage or generalized oxidative stress (Kamp et al. 2002).

## Conclusions

Our observation that XRCC1 deficiency sensitizes cells to the genotoxic effects of asbestos fibers provides the first mechanistic evidence to support the association between *XRCC1* polymorphisms and asbestos-related diseases. Cells exposed to asbestos fibers in the context of reduced XRCC1-mediated DNA repair are expected to accumulate higher levels of chromosomal DNA damage. Continued proliferation in the presence of biopersistent fibers could result in additional genomic alterations that promote malignant transformation. XRCC1 deficiency or reduced XRCC1 function may exacerbate genomic instability, increasing the risk of asbestos- associated malignancy.

## Supplemental Material

(11.2 MB) PDF
