# Supplementary material for: XRCC1 Deficiency Sensitizes Human Lung Epithelial Cells to Genotoxicity by Crocidolite Asbestos and Libby Amphibole
Source: Environ Health Perspect. 2010 Aug 12;118(12):1707–13. doi: 10.1289/ehp.1002312 (PMC3205592; doi:10.1289/ehp.1002312)
Supplement: (11.2 MB) PDF [file ehp.1002312.s001.pdf]

Supplemental Material

XRCC1 Deficiency Sensitizes Human Lung Epithelial Cells to Genotoxicity by Crocidolite Asbestos and Libby Amphibole

Jodie R. Pietruska, Tatiana Johnston, Anatoly Zhitkovich, Agnes B. Kane

1 Title Page

2 Table of Contents

3 Supplemental Material, Table 1. Size distribution of UICC crocidolite and Libby amphibole.

4 Supplemental Material, Figure 1. Spontaneous and damage-induced micronuclei in H460 cells.

5 Supplemental Material, Figure 2. Carcinogenic fibers activate the p53 pathway within 48 hours of exposure.

6 Supplemental Material, Figure 3. XRCC1 deficiency does not sensitize H460 cells to the growth inhibitory effects of fibers.

7 Supplemental Material, Figure 4. Nongenotoxic fibers and particulates do not induce nuclear bud formation.

8 References

Supplemental Material, Table 1. Size distribution of UICC crocidolite and Libby amphibole

	% of fibers in size range			% of fibers in size range	
Length (μm)	crocidolite asbestos	Libby amphibole	Diameter (μm)	crocidolite asbestos	Libby amphibole
0.1-1.0	46.4	12.6	0.03-0.10	12.4	5.3
1.1-5.0	44.8	38.5	0.11-0.25	56.6	21.1
5.1-8.0	3.8	23.1	0.26-0.50	18.6	36.1
8.1-10.0	0.9	10.4	0.51-1.00	9.7	26.3
10.1-20.0	2.4	11.6	1.01-1.50	0.9	9.0
≥ 20.1	1.7	3.6	1.51-2.5	1.8	2.3

Fiber size distributions were determined by transmission electron microscopy using the methods described in (Moalli 1987).

Supplemental Material, Figure 1: Spontaneous and damage-induced micronuclei in H460 cells

A

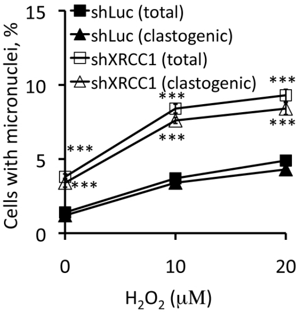

B

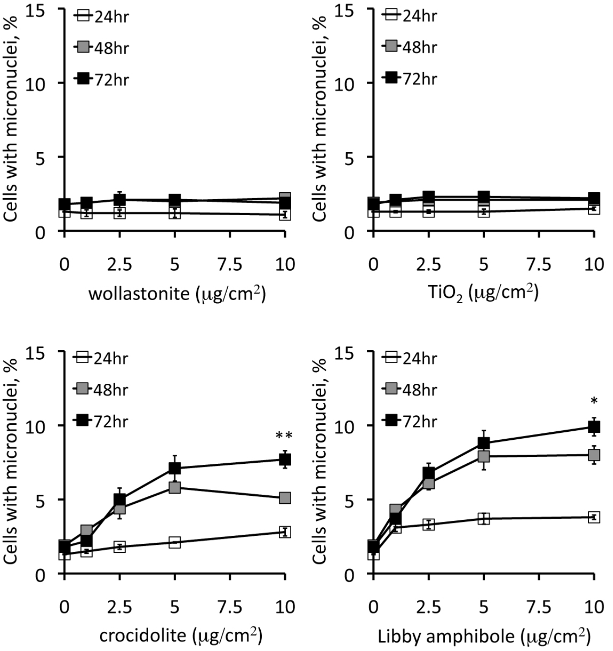

(A) XRCC1 deficiency increases frequencies of both spontaneous and H_2O_2-induced micronuclei in H460 cells.
Micronuclei in shLuc and shXRCC1 were scored 48 hours after a 1-hour exposure to H_2O_2. Filled symbols, shLuc; open symbols, shXRCC1; squares, total micronuclei; triangles, clastogenic micronuclei. Means ± SE of three replicates are shown. *, $P<0.05$, **, $P<0.01$, ***, $P<0.001$ compared with shLuc, one-way ANOVA.

(B) Formation of micronuclei by carcinogenic fibers is maximal at 5μg/cm^2.
H460 cells were exposed to 0-10μg/cm^2 particles or fibers for 24-72 hours and total micronuclei were scored in 1000 interphase cells per replicate. Means ± SE of three replicates are shown, *, $P<0.05$, **, $P<0.01$, ***, $P<0.001$ compared to 48hr, one-way ANOVA.

Supplemental Material, Figure 2. Carcinogenic fibers activate the p53 pathway

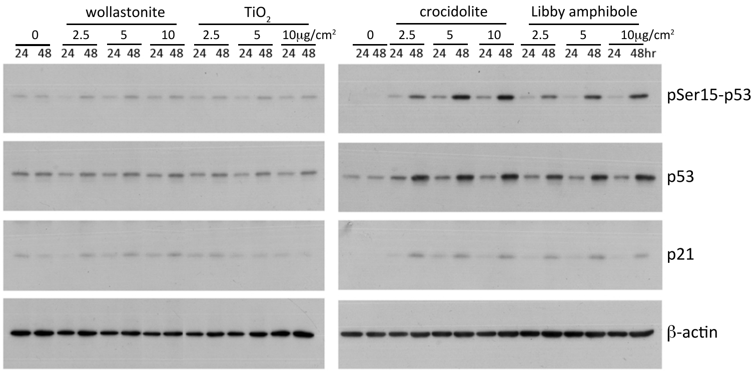

Western blot of shLuc cells exposed to 0-10μg/cm² wollastonite, TiO_2, crocidolite, or Libby amphibole for 24 hours or 48 hours. Exposure to crocidolite asbestos or Libby amphibole, but not wollastonite or TiO_2, induces p53 stabilization and upregulation of p21. β-actin was included as a loading control.

Supplemental Material, Figure 3: XRCC1 deficiency does not sensitize H460 cells to the growth inhibitory effects of fibers

A

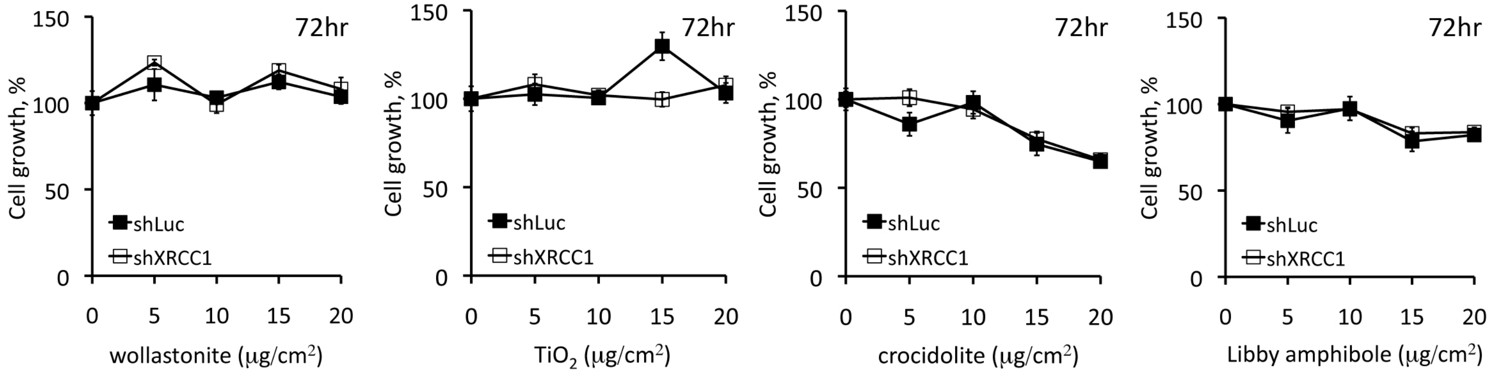

B

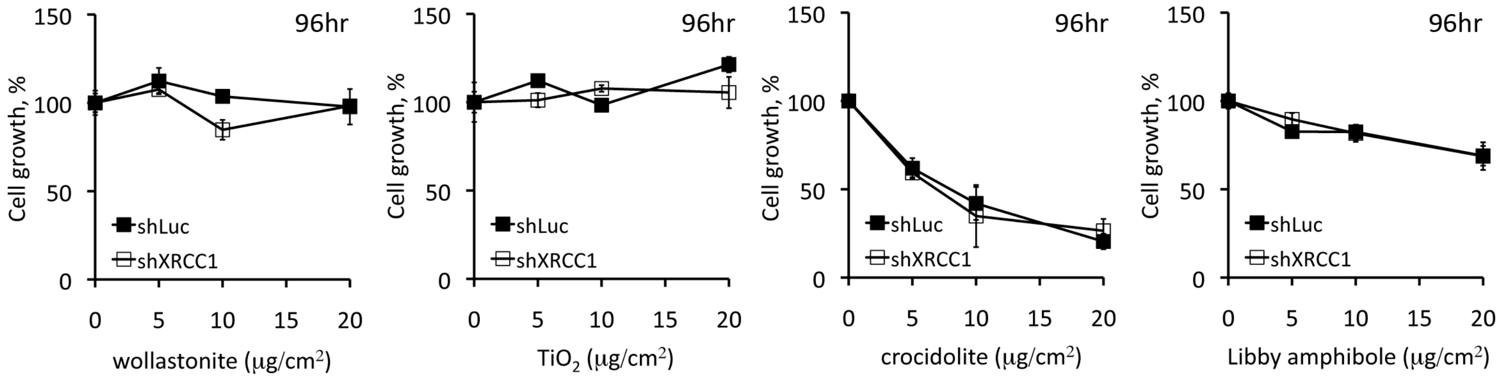

XRCC1 deficiency does not enhance growth inhibition by carcinogenic fibers at doses greater than 5µg/cm^2. shLuc (filled squares) and shXRCC1 (open squares) were exposed to 0-20µg/cm^2 fibers for (A) 72 hours or (B) 96 hours. Means ± SE of four replicates are shown.

Supplemental Material, Figure 4: Nongenotoxic fibers and particulates do not induce nuclear bud formation

A

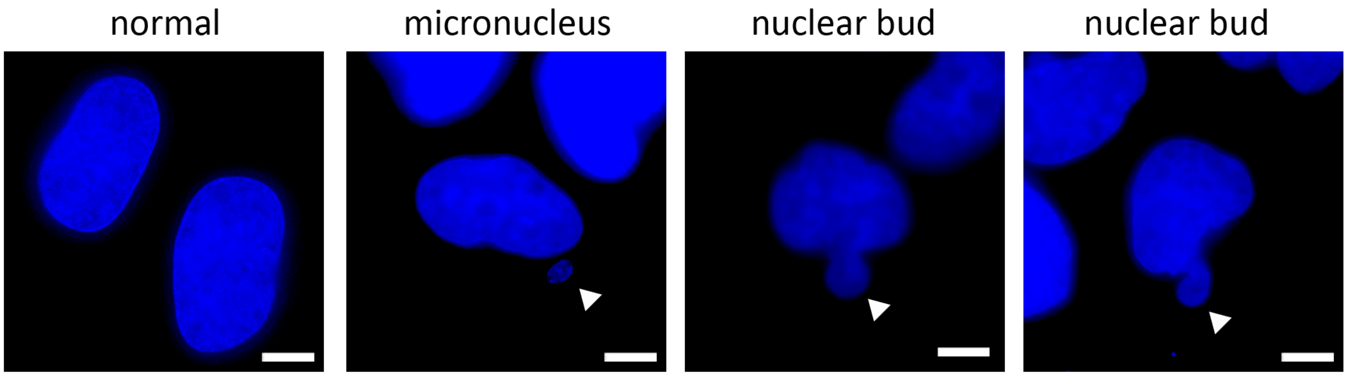

B

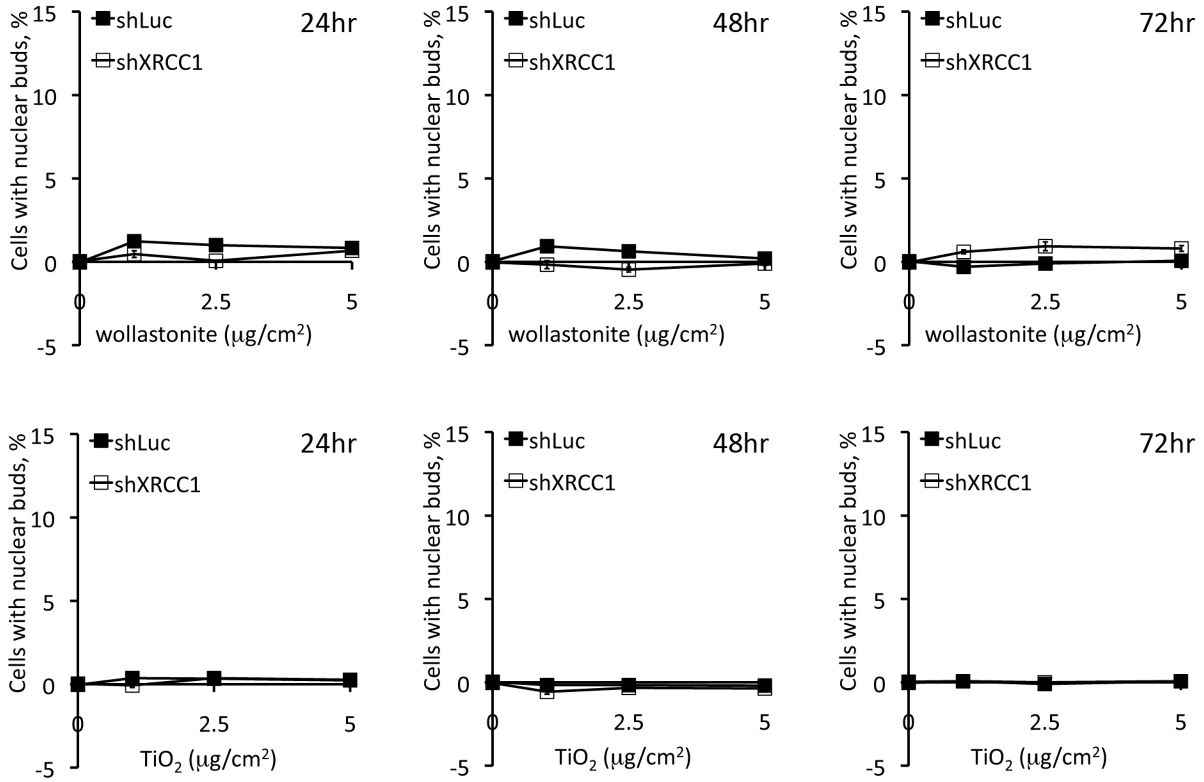

(A) Representative nuclear staining of H460 cells with no nuclear aberrations (normal), a micronucleus, or a nuclear bud. Scale bar, 20μm.
(B) Nuclear buds were scored in shLuc (filled squares) and shXRCC1 (open squares) exposed for 24, 48, or 72 hours to wollastonite or TiO_2. Means ± SE of three replicates are shown, in which the percentage of spontaneous nuclear buds has been subtracted. *, $P<0.05$, **, $P<0.01$, ***, $P<0.001$ compared with shLuc, one-way ANOVA.

References

Moalli PA, MacDonald JL, Goodglick LA, Kane AB. 1987. Acute injury and regeneration of the mesothelium in response to asbestos fibers. American Journal of Pathology 128(3):4426-445.
